# Supplementary material for: Resolving the full spectrum of human genome variation using Linked-Reads
Source: Genome Res. 2019 Apr;29(4):635–45. doi: 10.1101/gr.234443.118 (PMC6442396; doi:10.1101/gr.234443.118)
Supplement: Supplemental Material [file supp_gr.234443.118_Supplemental_longranger-2.2.zip › longranger-2.2.tar.bz2!/lr2.2-public/lib/stan/cmdstan/stan/doc/api/html/math_2functions_2if__else_8hpp_source.html]

Stan: src/stan/math/functions/if\_else.hpp Source File


|  |  |
| --- | --- |
|  | Stan  2.5.0  probability, sampling & optimization |


- Main Page
- Related Pages
- Namespaces
- Classes
- Files

- File List
- File Members

All Classes Namespaces Files Functions Variables Typedefs Enumerations Enumerator Friends Macros Pages

- src
- stan
- math
- functions

if\_else.hpp

Go to the documentation of this file.

1 #ifndef STAN\_\_MATH\_\_FUNCTIONS\_\_IF\_ELSE\_HPP

2 #define STAN\_\_MATH\_\_FUNCTIONS\_\_IF\_ELSE\_HPP

3

4 #include <boost/math/tools/promotion.hpp>

5

6 namespace stan {

7  namespace math {

8

23  template <typename T\_true, typename T\_false>

24  inline typename boost::math::tools::promote\_args<T\_true,T\_false>::type

25  if\_else(const bool c, const T\_true y\_true, const T\_false y\_false) {

26  return c ? y\_true : y\_false;

27  }

28

29  }

30 }

31

32 #endif

stan::math::if\_else

boost::math::tools::promote\_args< T\_true, T\_false >::type if\_else(const bool c, const T\_true y\_true, const T\_false y\_false)

Return the second argument if the first argument is true and otherwise return the second argument...

**Definition:** if\_else.hpp:25


---

[ Stan Home Page ]

*© 2011–2014,
Stan Development Team.*
